# Iron induces B cell pyroptosis through Tom20–Bax–caspase–gasdermin E signaling to promote inflammation post-spinal cord injury

**DOI:** 10.1186/s12974-023-02848-0

**Published:** 2023-07-22

**Authors:** Chengjie Wu, Lining Wang, Sixian Chen, Lei Shi, Mengmin Liu, Pengcheng Tu, Jie Sun, Ruihua Zhao, Yafeng Zhang, Jianwei Wang, Yalan Pan, Yong Ma, Yang Guo

**Affiliations:** 1 Department of Traumatology and Orthopedics, Affiliated Hospital of Nanjing University of Chinese Medicine, Nanjing, China; 2 Laboratory of New Techniques of Restoration and Reconstruction, Institute of Traumatology and Orthopedics, Nanjing University of Chinese Medicine, Nanjing, China; 3 School of Chinese Medicine, School of Integrated Chinese and Western Medicine, Nanjing University of Chinese Medicine, Nanjing, China; 4 Laboratory of Chinese Medicine Nursing Intervention for Chronic Diseases, Nanjing University of Chinese Medicine, Nanjing, China; 5 Jiangsu CM Clinical Innovation Center of Degenerative Bone and Joint Disease, Wuxi TCM Hospital Affiliated to Nanjing University of Chinese Medicine, Wuxi, China

**Keywords:** Spinal cord injury, B cells, Cellular pyroptosis, Immune inflammatory response, Iron, Tom20

## Abstract

**Background:**

Immune inflammatory responses play an important role in spinal cord injury (SCI); however, the beneficial and detrimental effects remain controversial. Many studies have described the role of neutrophils, macrophages, and T lymphocytes in immune inflammatory responses after SCI, although little is known about the role of B lymphocytes, and immunosuppression can easily occur after SCI.

**Methods:**

A mouse model of SCI was established, and HE staining and Nissl staining were performed to observe the pathological changes. The size and morphology of the spleen were examined, and the effects of SCI on spleen function and B cell levels were detected by flow cytometry and ELISA. To explore the specific mechanism of immunosuppression after SCI, B cells from the spleens of SCI model mice were isolated using magnetic beads and analyzed by 4D label-free quantitative proteomics. The level of inflammatory cytokines and iron ions were measured, and the expression of proteins related to the Tom20 pathway was quantified by western blotting. To clarify the relationship between iron ions and B cell pyroptosis after SCI, we used FeSO_4_ and CCCP, which induce oxidative stress to stimulate SCI, to interfere with B cell processes. siRNA transfection to knock down Tom20 (Tom20-KD) in B cells and human B lymphocytoma cell was used to verify the key role of Tom20. To further explore the effect of iron ions on SCI, we used deferoxamine (DFO) and iron dextran (ID) to interfere with SCI processes in mice. The level of iron ions in splenic B cells and the expression of proteins related to the Tom20–Bax–caspase–gasdermin E (GSDME) pathway were analyzed.

**Results:**

SCI could damage spleen function and lead to a decrease in B cell levels; SCI upregulated the expression of Tom20 protein in the mitochondria of B cells; SCI could regulate the concentration of iron ions and activate the Tom20–Bax–caspase–GSDME pathway to induce B cell pyroptosis. Iron ions aggravated CCCP-induced B cell pyroptosis and human B lymphocytoma pyroptosis by activating the Tom20–Bax–caspase–GSDME pathway. DFO could reduce inflammation and promote repair after SCI by inhibiting Tom20–Bax–caspase–GSDME-induced B cell pyroptosis.

**Conclusions:**

Iron overload activates the Tom20–Bax–caspase–GSDME pathway after SCI, induces B cell pyroptosis, promotes inflammation, and aggravates the changes caused by SCI. This may represent a novel mechanism through which the immune inflammatory response is induced after SCI and may provide a new key target for the treatment of SCI.

**Supplementary Information:**

The online version contains supplementary material available at 10.1186/s12974-023-02848-0.

## Background

After experiencing a spinal cord injury (SCI), the central nervous system rarely recovers completely, and there is a high probability of disability, which places a heavy burden on the family of patients and society as a whole [1]. SCI comprises both primary and secondary injuries. The primary injury involves the destruction of spinal cord tissue, which is often difficult to mitigate in time, whereas secondary injury is characterized by the subsequent disturbance of microcirculation, immune-mediated inflammatory reactions, apoptosis, and the formation of glial scars; the ability to limit such damage is a key factor in the treatment of SCI [2]. The inflammatory response that occurs in the early stage of SCI largely determines the subsequent progression and extent of the secondary injury, and patients with SCI may experience a chronic inflammatory state for a long period of time, affecting the recovery of neurological function [3–5]. Therefore, many studies have suggested that controlling inflammation is beneficial for promoting recovery after SCI [6–9], and there are many promising pharmacological methods for reducing inflammation [10–12].

Many types of immune cells are involved in the inflammatory response after SCI, including neutrophils, macrophages, microglia, astrocytes, B lymphocytes, and T lymphocytes, which can infiltrate the injured spinal cord. However, the composition and role of immune cell populations change depending on the stages of inflammation and the signals present in the microenvironment [13–15]. The regulation of immune function depends on the activity of sympathetic nerves, and the loss of sympathetic innervation of lymphoid organs after SCI leads to atrophy of the spleen and lymphocytopenia. In contrast, SCI reduces or completely disrupts the transmission of nerve signals to the hypothalamus from the adrenal gland; this is accompanied by abnormal feedback of the hypothalamic–pituitary–adrenal axis, which further impairs the function of the spleen and reduces the number of lymphocytes. The immunosuppressive syndrome induced by SCI has been described previously [16]. Some studies have shown increased activation of B cells after SCI, and nerve repair is induced directly by the production of specific antibodies [17]. Mice lacking B cells exhibit enhanced recovery of motor function after SCI [18]. However, other studies have shown that immunoglobulin M knockout (IgM-KO) mice exhibit a significant delay in neurobehavioral recovery after SCI [19]. At present, the relationship between SCI and B cell function remains controversial, and the underlying mechanism driving such changes remains unclear.

In this study, we first aimed to determine the effect of SCI on spleen function in animal experiments and found that SCI could downregulate the levels of IgM and the number of B cells. We used 4D label-free quantitative proteomics to analyze the differential changes in proteins in B cells after SCI and found that the model group exhibited significantly higher expression of Tom20, the levels of which were related to oxidative stress and the immune inflammatory response, and the molecular action sites were located within the mitochondria and at the cell membrane. We subsequently aimed to quantify the levels of iron ions in B cells and to determine the mechanism responsible for the changes. We found that the concentration of iron ions in B cells increased abnormally after SCI, and iron ions induced B cell pyroptosis through the Tom20–Bax–caspase–gasdermin E (GSDME) pathway to aggravate the inflammatory response after SCI both in vivo and in vitro, which may represent a novel immune inflammatory response mechanism induced by SCI.

## Methods

### Animals

For all animal experiments, female C57BL/6 mice (6–8 weeks) were purchased from Qinglong Mountain Animal Breeding Farm in Nanjing (license number: SYXK (Su) 2019-0010). The mice were raised in a specific-pathogen-free environment and were allowed free access to food and water at any time. All experimental procedures involving animals were approved by the Animal Ethics Committee of Nanjing University of Chinese Medicine (201912A023) and were conducted in accordance with the guidelines of the Ministry of Public Health of China on the care and use of laboratory animals.

### Cells

Human B lymphocytoma cells (Ramos) were purchased from Procell Life Science & Technology Co., Ltd. (CL-0483) and cultured in Roswell Park Memorial Institute (RPMI)-1640 medium containing 10% fetal bovine serum (FBS) and 1% penicillin/streptomycin. Mouse spinal cord neurons were purchased from Procell Life Science & Technology Co., Ltd. (CP-M178) and cultured in the special neuron culture medium (CM-M178) provided by the company. For primary B cell collection, 10 C57BL/6 mice were sacrificed under excessive anesthesia, and the spleens were removed and placed on a 200-μm mesh cell filter. The spleens were ground using a 5 mL syringe, and RPMI-1640 medium was added simultaneously. After collecting the filtrate, lymphocytes were separated using a commercial lymphocyte separation kit (Solarbio, P8860), then centrifuged at 300 × *g* for 10 min at 4 ℃. The supernatant was discarded, and the cells were resuspended. A 10 μL volume of CD19 magnetic beads (Miltenyi Biotec, 130-121301) was added to the solution and incubated for 10 min at 4 ℃. Magnetic beads were separated using a sorting column and magnetic equipment. After centrifugation, the supernatant was resuspended, and the B cells were cultured in an incubator.

### SCI model

The SCI model was established in C57BL/6 mice using a modified A11en method [20]. Briefly, mice were anesthetized via an intraperitoneal injection of 1% pentobarbital sodium (80 mg/kg). After iodophor disinfection, the skin was cut longitudinally along the spine with a scalpel, the T9–T11 laminae were exposed, a laminectomy was performed at T10, and a spinal cord percussion device was used to strike the spinal cord at T10. Immediate release of tension in both hind limbs was indicative of the successful establishment of the model. After surgery, the mice were placed on a cotton pad until full recovery from anesthesia. The bladder was emptied manually daily to prevent urinary tract infection until the mice were able to urinate independently. Mice in the sham group underwent the same procedure, but without any impact to the spinal cord.

### Drug intervention

For animal experiments, mice in the DFO group received intraperitoneal injection of 100 mg/kg deferoxamine mesylate salt (Yuanye, S61301), and mice in the ID group were injected with iron dextran (Yuanye, S51662) at a dose of 10 mg/kg after modeling. Mice in the model and sham operation groups were injected with the same volume of normal saline, and the indexes were detected 3 days later [21]. For the cell experiments, 20 μM CCCP (Yuanye, S85757) and 100 μM FeSO_4_ (Yuanye, S50639) were used to interfere with cellular processes of SCI [22].

### BMS scoring

BMS scoring was conducted blinded to the conditions. One person randomly selected the mice and was responsible for recording the results, while another individual was responsible for observing the locomotor activity of the mice to assign the score. During the evaluation, the animals were placed on open, flat ground, and limb movements were observed and recorded.

### Oblique board test

Different groups of mice were placed on a rectangular ramp perpendicular to the longitudinal axis of the ramp. The height of the board was raised, and the maximum angle at which the rats stayed on the board for more than 5 s was recorded. Each mouse was tested three times, and the average value was taken as the final result.

### Neuroelectrophysiological detection

Three days after SCI, the motor evoked potentials and electromyography data of mice in each group were collected by information biological signal acquisition and processing system (TaiMeng, BL-420N). The stimulation intensity was 1 V and the stimulation frequency was 0.3 Hz. The signals were collected and stored, and at least 12 waveforms were collected each time.

### Hematoxylin and Eosin (HE), Nissl, and Prussian blue staining

Paraffin-embedded sections (3–4 μm in thickness) of tissue surrounding the lesion site in the spinal cord and spleen were stained using an HE staining kit (Servicebio, G1005), a Nissl staining kit (Servicebio, G1036), and Prussian blue staining kit (Servicebio, G1029). After the addition of neutral gum to the spinal cord slices, the slides were covered, and the sections were observed under a microscope.

### Enzyme-linked immunosorbent assay (ELISA) and iron ion detection

Serum samples and cell supernatants were collected and analyzed using commercially available ELISA kits for mice, including IL-1β (mlbio, ml301814), IL-6 (mlbio, ml063160), MCP-1 (mlbio, ml037840), IgG (mlbio, ml037601), IgM (mlbio, ml063597), and TNF-α (mlbio, ml002095) kits. The absorbance of each well was measured at a 450-nm wavelength. The iron ion concentration in the samples was detected using an iron ion colorimetric detection kit (Applygen, E1042), and the absorbance of each well was measured sequentially at a 560-nm wavelength.

### Immunofluorescence and TUNEL staining

For paraffin sections, antigen repair was performed after dewaxing, and blocking was performed using 5% BSA for 30 min at room temperature. The sections were incubated with primary antibodies, CD19 (1:200, Servicebio, GB11061-1) and NeuN (1:200, Abcam, ab104224), at 4 ℃ overnight and washed thrice with PBS. The secondary antibody, Alexa-594 (1:500, Abcam, ab150080), was then added and incubated at room temperature for 1 h, followed by washing with PBS thrice. For the TUNEL staining, TUNEL Apoptosis Detection Kit (Yeasen, 40306ES50) was used. After Dapi re-staining, PBS was used to wash, and anti-fluorescence quenching agent was added. The sections were observed under fluorescence inverted microscope.

### Flow cytometry

Splenic lymphocytes were collected for flow cytometric analysis. After centrifugation at 300 × *g* for 10 min at 4 ℃, the supernatant was discarded, and the cells were re-suspended. After adding 10 µL of an Fc receptor blocker (Miltenyi Biotec, 130-09257575), the cells were incubated at 4 °C for 10 min. Subsequently, an allophycocyanin-conjugated antibody, CD19-APC (BD, 550992), and a phycoerythrin-conjugated antibody, CD138-PE (BioLegend, 142504), were added at a volume of 1 µL each, and the cells were incubated at 4 °C for 30 min in the dark. After washing, centrifugation, and resuspension, the samples were analyzed using flow cytometry (Merck Millipore, USA) and IDEAS software (Merck Millipore, USA).

### 4D label-free quantitative proteomic analysis

Splenic B cells were extracted 3 days after SCI using a magnetic bead sorting technique, and 4D label-free quantitative proteomic analysis was performed by Shanghai Genechem Co., Ltd. The main experimental procedures included protein extraction, quantification, and detection, as well as filter-aided sample preparation (FASP) enzymatic hydrolysis, mass spectrometry detection, and data analysis. The detailed steps can be found in Additional file 1.

### CCK8 assessment of cell viability and LDH detection

To assess cell viability, a CCK8 kit (Yeasen, 40203ES76) was used, and the absorbance of each well was measured sequentially at a 450-nm wavelength. The release of LDH was quantified using an LDH kit (Beyotime, C0017), and the absorbance of each well was measured sequentially at a 490-nm wavelength.

### Cell transfection

siRNA-Mate transfection reagent was used to transfect siRNA oligonucleotides into cells to achieve gene silencing of Tom20. The sequence of the siRNA oligonucleotide applied to mouse B cells was as follows: small hairpin RNA (shRNA)-Tom20, 5ʹ-CCUGACAAAUGCCAUUGCUTT-3ʹ; the sequence of the siRNA oligonucleotide applied to human Ramos cells was as follows: shRNA-Tom20, 5ʹ-GGCUUCGAGAACGAAGAAATT-3ʹ; and the sequence of the siRNA oligonucleotide applied to the negative control was as follows: shRNA-negative control, 5ʹ-UUCUCCGAACGU GUCACGUTT-3 ʹ. All reagents were purchased from Suzhou GenePharma Co., Ltd.

### Adeno-associated virus (AAV) injection

We entrusted OBiO Technology (Shanghai) Corp., Ltd to construct the viral vector: the viral type was adeno-associated virus, the viral serotype was AAV2/8, and the name after construction was pAAV–U6-shRNA (Tomm20)–CMV–EGFP–WPRE. The shRNA sequence was as follows: GCAGTTCAGAAATTCTTCCTT. The shRNA sequence of the negative control was as follows: CCTAAGGTTAAGTCGCCCTCG. The vector was injected through the tail vein at a dose of 4 × 10^12^ gc/kg [23]. Three weeks later, the mouse SCI model was established.

### Western blot

Radioimmunoprecipitation assay (RIPA) lysate (containing 1% phosphatase and protease inhibitor) was added to a cell plate, the cells were dissociated on ice for 30 min, and centrifuged at 12,000 × *g* for 10 min at 4 °C. After the supernatant was collected, protein content was detected using the bicinchoninic acid (BCA) method. Upon addition of loading buffer, the supernatant was placed in a boiling water bath for 10 min. Precast gels (ACE, F15420Gel) were used for electrophoresis. The proteins were transferred to an activated polyvinylidene fluoride (PVDF) membrane, blocked with 5% skim milk for 2 h at room temperature, and rinsed with TBST. The primary antibodies used in this study were as follows: anti-cleaved-caspase 9 (1:1000, Affinity Biosciences, AF5240), anti-cleaved-caspase 3 (1:1000, Affinity, AF7022), anti-cytochrome C (1:1000, Affinity, AF0146), anti-GSDME-N (1:1000, Affinity, AF4016), anti-Tom20 (1:5000, Proteintech, 11802-1-AP), anti-Bax (1:10,000, Proteintech, 60267-1-Ig), and anti-β-actin (1:10,000, Proteintech, 66009-1-Ig). After incubation in primary antibody solution overnight at 4 °C, membranes were washed three times with TBST. The secondary antibodies used in this study were as follows: goat anti-rabbit horseradish peroxidase (HRP) (1:5000, Affinity, S0001) and goat anti-mouse HRP (1:5000, Affinity, S0002). After incubation at room temperature for 1.5 h, the membranes were washed three times in TBST. Enhanced chemiluminescent (ECL) reagent was added prior to the analysis using a gel imaging system (Tanon, 5200CE).

### Statistical analysis

Two-tailed unpaired Student’s *t* tests were used to determine whether significant differences existed between two groups. Differences among multiple groups were analyzed by one- or two-way analysis of variance (ANOVA), followed by Tukey’s post-hoc testing when appropriate. All statistical analyses were performed using GraphPad Prism version 8 software. Values are expressed as the mean ± SD. Statistical significance was set at *P* < 0.05.

## Results

### SCI can damage spleen function and lead to a decrease in B cell levels

**Fig. 1:**
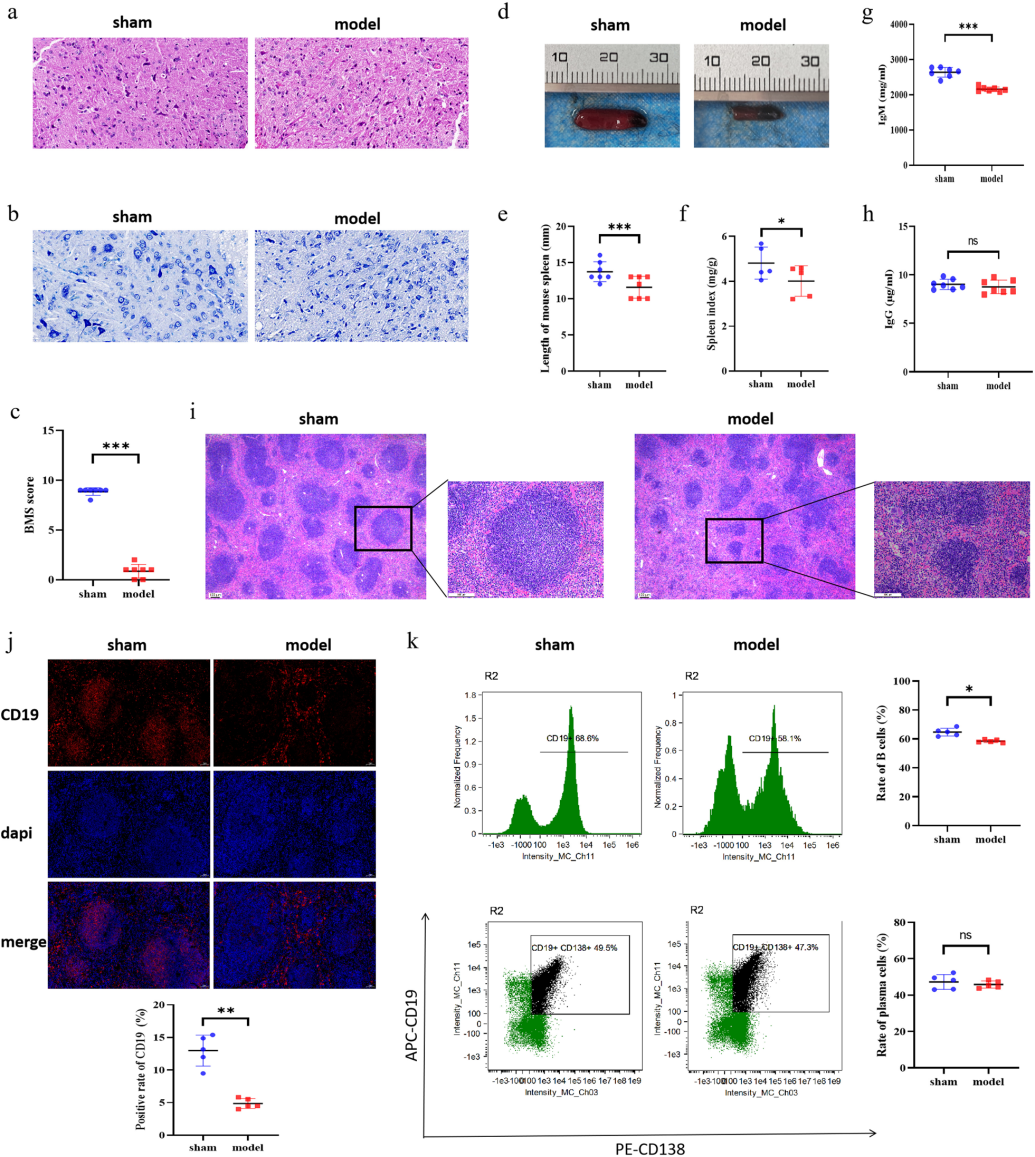
Effects of SCI on spleen function and B cell levels. **a**–**c** After the establishment of the SCI model in mice, motor function was evaluated based on the BMS score determined on the 3rd day. HE staining to assess overall structure and inflammatory infiltration and Nissl staining to assess neuronal morphology and the intracellular condition were performed on injured spinal cord tissue. **d**–**f** Spleens of mice collected three days after SCI were examined, and the length and organ index of the spleens were compared among the different groups. **g**, **h** Three days after SCI, mouse serum samples were collected to determine and compare the expression levels of IgG and IgM in the different groups via ELISA. **i**, **j** Spleen of mice 3 days after SCI was stained with HE and detected by immunofluorescence. The results showed that CD19^+^ was red, dapi was blue, and merge was combined. **k** Spleens of mice were collected 3 days after SCI, and the lymphocytes were isolated and detected by flow cytometry (B cells were CD19^+^, and plasma cells were CD19^+^ and CD138^+^). All data are expressed as the mean ± SD (*n* ≥ 3 replicates per group). ns *P* > 0.05, * *P* < 0.05, *** *P* < 0.001. *SCI* spinal cord injury, *BMS* Basso Mouse Scale, *HE* hematoxylin & eosin, *IgG* immunoglobulin G, *IgM* immunoglobulin M, *ELISA* enzyme-linked immunosorbent assay, *CD* cluster of differentiation, *SD* standard deviation, *ns* not significant

First, we established a mouse model of SCI, and the effects of SCI in mice were determined based on the Basso Mouse Scale for locomotion (BMS) score measured on the third day after injury. HE and Nissl staining were conducted to observe the pathological changes present in the cross-sectional areas of the injured spinal cord. The staining revealed that the nerve fibers in the model group were disordered, with extensive inflammatory cell infiltration and partial neuronal necrotic pyknosis (Fig. 1a, b), which was consistent with previous research findings [12]. The present results confirmed the successful establishment of the model (Fig. 1c). We examined the size and morphology of the spleen of mice and found that the length of the spleen in the model group became smaller and darker in color, and the spleen index of the model group also decreased significantly (Fig. 1d–f). The ELISA results revealed a downregulation in the levels of serum IgM in the model group, although no significant change was observed in immunoglobulin G (IgG) levels (Fig. 1g, h), which may have been related to early immune regulation. The pathological changes of spleen in mice were observed by HE staining and immunofluorescence. White pulp in the model group was atrophied, the structure was destroyed, and the number of B cells decreased (Fig. 1i, j). In addition, the flow cytometry results demonstrated a decrease in the proportion of B cells in the spleens of mice in the model group (Fig. 1k). Collectively, these results suggested that SCI can damage spleen function, and the immunosuppression was similar to that observed in previous studies [16].

### SCI upregulates the expression of Tom20 protein in mitochondria of B cells

**Fig. 2:**
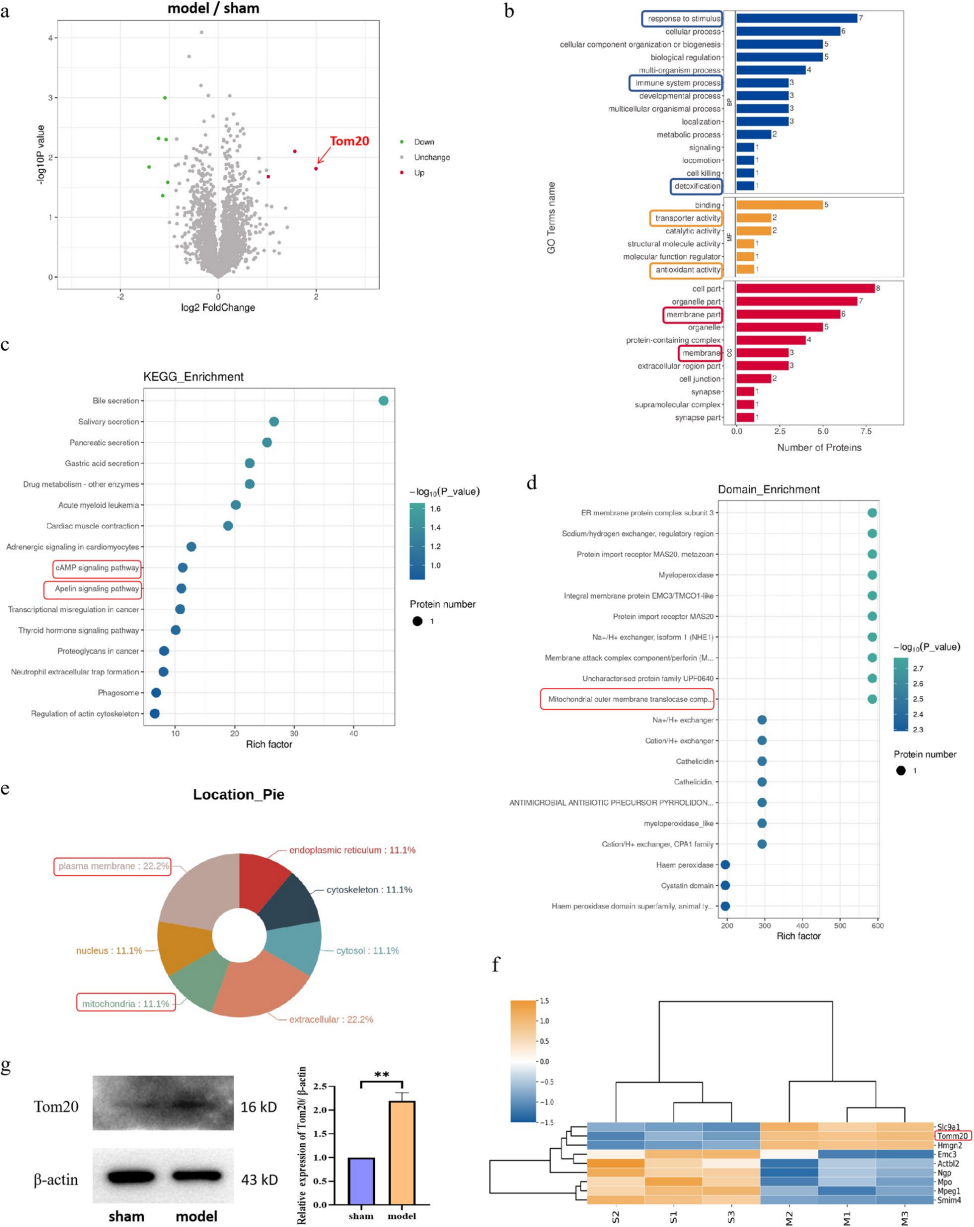
Protein expression levels in B cells from the spleens of mice after SCI were analyzed by 4D label-free quantitative proteomics. **a** In the significant difference analysis of the quantitative results, we first selected at least half of the repeated experimental data in the sample group for statistical analysis. Proteins with expression differences of more than twofold and a *P* ≤ 0.05 are regarded as differentially expressed proteins. The results of the differential protein screening are displayed in the form of a volcanic map, with red spots indicating upregulated proteins (red arrow indicates Tom20), green spots indicating downregulated proteins, and gray indicating proteins with no difference in expression. **b** GO functions of all differentially expressed proteins were analyzed using Blast2Go software. Blue indicates involvement in biological processes, orange indicates a role in cell composition, red indicates a role in molecular function, and the corresponding box represents the cell function of interest. **c** Taking the Kyoto Encyclopedia of Genes and Genomes (KEGG) pathway as a unit and all qualitative proteins as the background, the significance level of protein enrichment in each pathway was analyzed and calculated by Fisher’s exact test to determine the metabolic and signal transduction pathways that were significantly affected. The red box shows the cellular pathway of interest. **d** Functional domains of differentially expressed proteins were analyzed using the InterPro database, and the annotated results of differential protein domains were enriched. The red box indicates the domain of interest. **e** The subcellular localization of differentially expressed proteins was analyzed using WoLF PSORT software. The red box indicates the subcellular location of interest. **f** In the clustering analysis, a clustering algorithm was used to classify the two dimensions of samples and variables. The red box indicates the Tom20 protein. **g** Western blotting was used to verify the expression of Tom20 in B cells from the spleens of mice after SCI. The data of Western blotting are expressed as the mean ± SD (*n* ≥ 3 replicates per group). ** P < 0.01. *SCI* spinal cord injury, *GO* gene ontology, *KEGG* Kyoto Encyclopedia of Genes and Genomes, *SD* standard deviation

To explore the specific mechanism of immunosuppression after SCI, B cells from the spleens of SCI model mice were isolated using magnetic beads and analyzed by 4D label-free quantitative proteomics. The volcanic map revealed that three proteins were upregulated and six proteins were downregulated in the model group; of these, Tom20 exhibited the greatest fold-change (Fig. 2a). The results of the gene ontology (GO) and Kyoto Encyclopedia of Genes and Genomes (KEGG) analyses showed that these differentially expressed proteins were related to oxidative stress and the immune inflammatory response (Fig. 2b, c). The results of the domain enrichment and subcellular localization analyses revealed that the molecular sites were mainly located within the mitochondria and at the cell membrane (Fig. 2d, e). The cluster analysis suggested that the upstream and downstream proteins related to Tom20 may include solute carrier family 9 member A1 (Slc9a1), high mobility group nucleosomal binding domain 2 (HMGN2), and endoplasmic reticulum membrane protein complex subunit 3 (Emc3), among others (Fig. 2f). Finally, we verified by western blotting that the expression of Tom20 in the model group was abnormally upregulated (Fig. 2g), suggesting that this protein is worthy of further exploration.

### SCI can regulate the concentration of iron ions and activate the Tom20–Bax–caspase–GSDME pathway to induce B cell pyroptosis

**Fig. 3:**
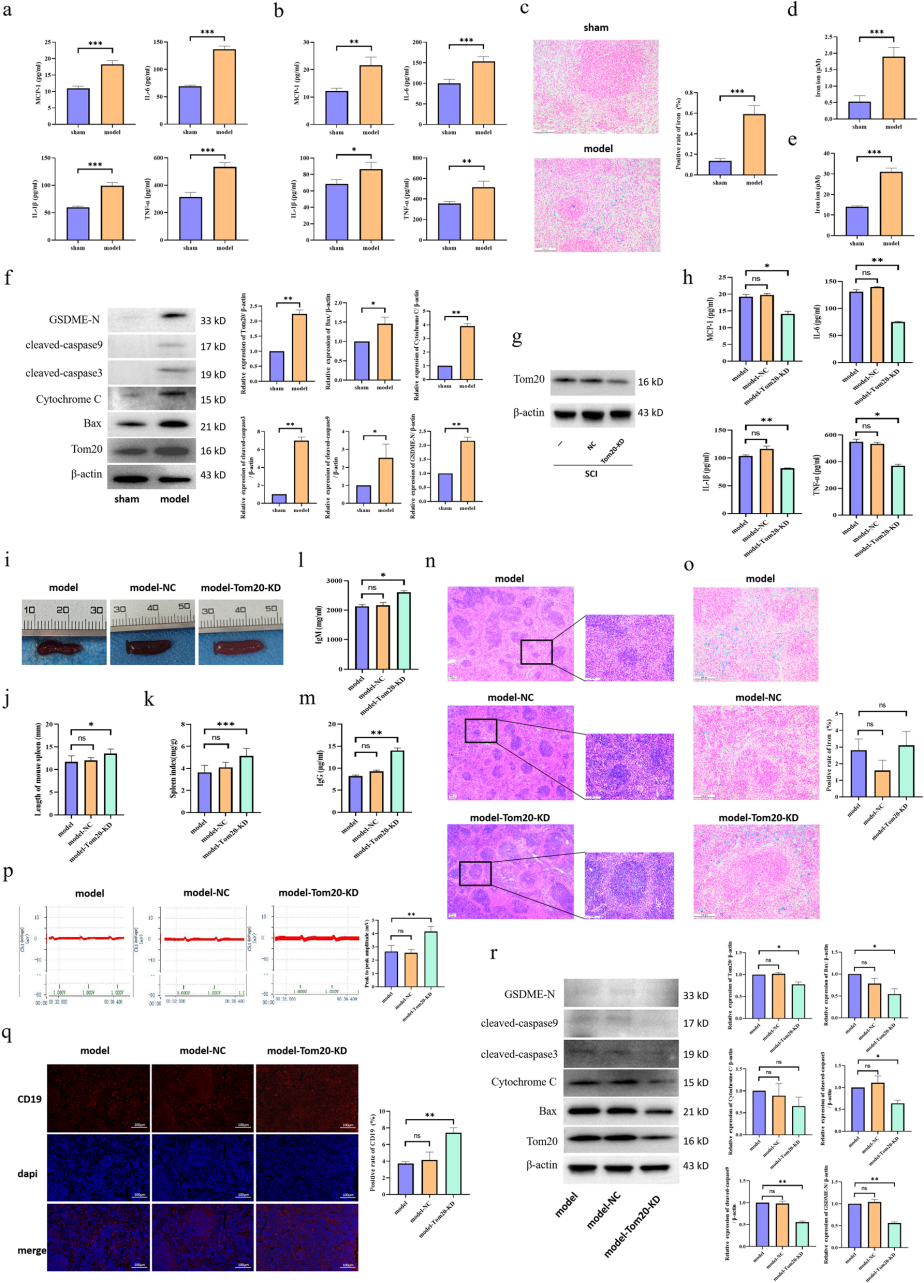
Pyroptosis induced by iron accumulation may occur in splenic B cells after SCI. **a** Three days after SCI, serum samples were collected for quantification of the protein expression levels of MCP-1, IL-1β, IL-6, and TNF-α by ELISA. **b** Three days after SCI, injured spinal cord homogenates were collected for quantification of the protein expression levels of MCP-1, IL-1β, IL-6, and TNF-α by ELISA. **c** Three days after SCI, the spleen was stained with Prussian blue to detect the level of iron ion. **d** Three days after SCI, serum samples were collected to quantify the concentration of iron ions. **e** Three days after SCI, B cell lysates were collected to quantify the concentration of iron ions. **f** Detection of Tom20-Bax-caspase-GSDME pathway-related protein expression in B cells by western blot. **g** The knock-down efficiency of AAV on Tom20 in B cells was verified. **h**, **l**, **m** The expression levels of MCP-1, IL-1 β, IL-6, TNF- α, IgG and IgM in serum of different groups were detected by ELISA. **i**–**k** Three days after SCI, the spleens of mice were observed, and the spleen length and organ index of different groups were compared. **n**, **o**, **q** The spleen was taken for HE staining, Prussian blue staining, and immunofluorescence. CD19^+^ was red, dapi was blue, and merge was combined. **p** Detection of nerve evoked potentials in different groups of mice. **r** Detection of the expression of Tom20-Bax-caspsae-GSDME pathway-related proteins in different groups of B cells. All data are expressed as the mean ± SD (*n* ≥ 3 replicates per group). ns *P* > 0.05, * P < 0.05, ** P < 0.01, *** *P* < 0.001. *SCI* spinal cord injury, *MCP-1* monocyte chemoattractant protein 1, *IL-1β* interleukin 1 beta, *IL-6* interleukin 6, *TNF-α* tumor necrosis factor alpha, *ELISA* enzyme-linked immunosorbent assay, *GSDME* gasdermin E, *SD* standard deviation, *ns* not significant

It is well-known that the levels of inflammatory cytokines are upregulated after SCI. Consistent with previous findings, our results also showed that the levels of inflammatory cytokines [monocyte chemoattractant protein 1 (MCP-1), interleukin 1 beta (IL-1β), interleukin six (IL-6), and tumor necrosis factor alpha (TNF-α)] were significantly upregulated in the serum and injured spinal cord samples of SCI model mice (Fig. 3a, b). We speculate that these changes are related to the downregulation of B cell levels. Some studies have shown that iron accumulation increases significantly after SCI [24]. We not only found that the level of iron ions was upregulated in serum samples after SCI, but we also observed iron accumulation in B cells of the spleen (Fig. 3c–e). Another study suggested that iron ions could induce cellular pyroptosis through the Tom20 pathway [22]. We speculated that B cell pyroptosis might also occur after SCI, which could lead to aggravation of inflammatory responses. Therefore, we quantified the expression of proteins related to the Tom20 pathway by western blotting and found that the Tom20–Bax–caspase–GSDME pathway was activated to induce B cell pyroptosis after SCI (Fig. 3f), possibly as a result of iron accumulation. We further used AAV to knock down the Tom20 gene in mice, and verified by western blotting that Tom20-KD mice were successfully established (Fig. 3g). As expected, in Tom20-KD group, the level of inflammatory factors decreased significantly (Fig. 3h), spleen function recovered significantly (Fig. 3i–n, q), and neuroelectrophysiology improved (Fig. 3p), but no significant difference was observed in iron content (Fig. 3o). Western blotting revealed that Tom20–Bax–caspsae–GSDME pathway was significantly inhibited in Tom20-KD group, and B cell pyrogenesis was reduced (Fig. 3r).

### Iron ions aggravate carbonyl cyanide m-chlorophenyl hydrazone (CCCP)-induced B cell pyroptosis by activating the Tom20–Bax–caspase–GSDME pathway

**Fig. 4:**
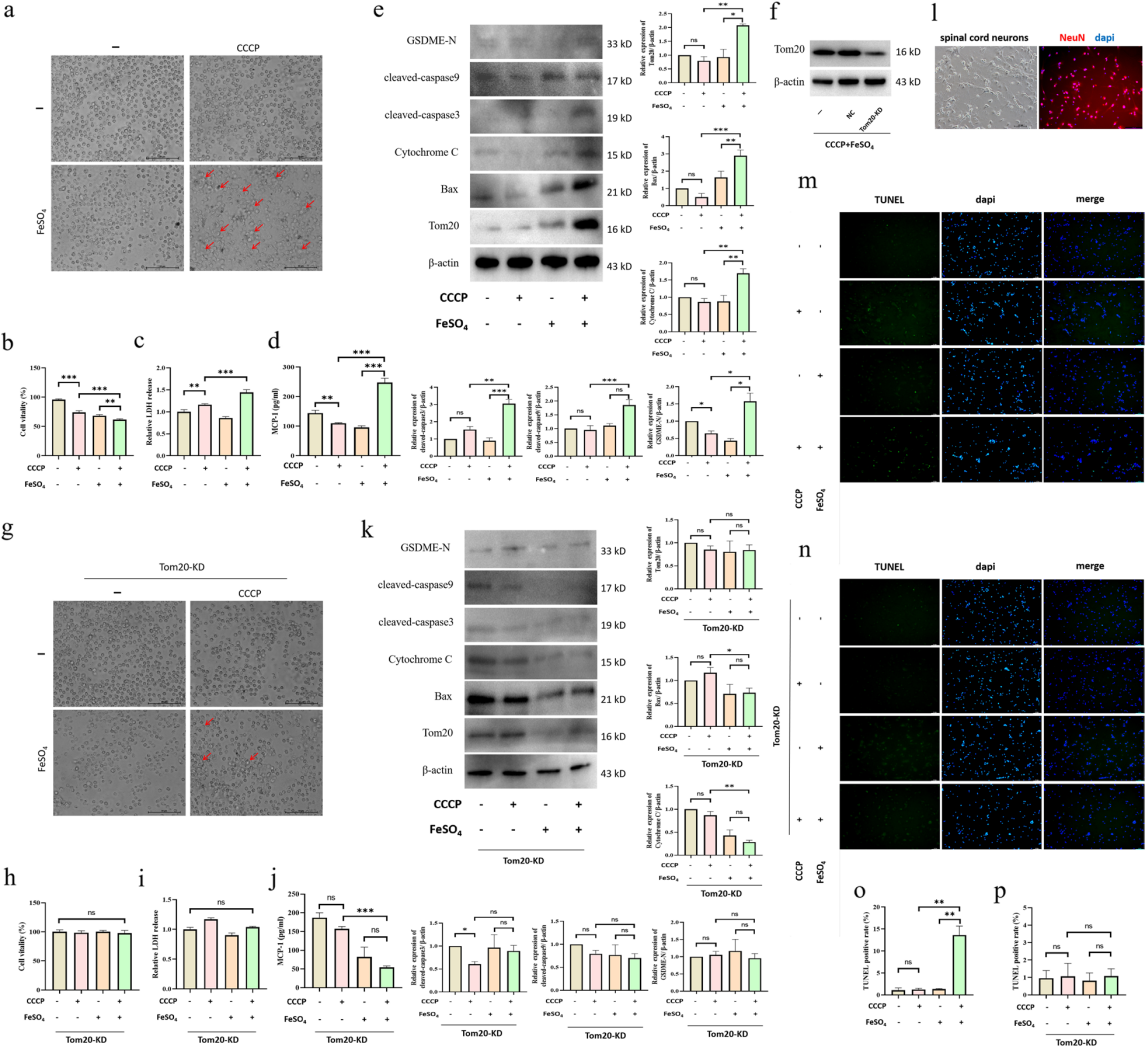
Effect of iron ions on CCCP-induced pyroptosis of B cells. **a** Interference of splenic B cells of mice was induced using FeSO_4_ and CCCP. After 24 h, the morphology of the B cells was observed. The red arrow indicates swollen B cells. **b** Detection of B cell viability by CCK8. **c** Detection of LDH release from B cell supernatants using an LDH kit. **d** Detection of the levels of MCP-1 in B cell supernatants by ELISA. **e** Detection of the expression levels of pyroptosis-related proteins in B cells by western blot. **f** After Tom20-KD via siRNA transfection, the expression of Tom20 was detected. **g** The morphology of B cells was observed after 24 h. The red arrow indicates swollen B cells. **h** Detection of B cell viability by CCK8 after TOM20-KD. **i** Detection of LDH release in B cell supernatants after Tom20-KD using an LDH kit. **j** Detection of the levels of MCP-1 in B cell supernatants after Tom20-KD via ELISA. **k** Western blot detection of the expression of pyroptosis-related proteins in B cells after Tom20-KD. **l** Morphological and immunofluorescence identification of spinal cord neurons in mice; NeuN was red. **m**–**p** After intervention, the supernatant of B cells was co-cultured with spinal cord neurons, and the apoptosis of neurons was observed by TUNEL staining. TUNEL was green, and dapi was blue. All data are expressed as the mean ± SD (*n* ≥ 3 replicates per group). ns *P* > 0.05, * *P* < 0.05, ** P < 0.01, *** *P* < 0.001. *CCCP* carbonyl cyanide m-chlorophenyl hydrazone, *CCK8* cell counting kit 8, *LDH* lactate dehydrogenase, *MCP-1* monocyte chemoattractant protein 1, *ELISA* enzyme-linked immunosorbent assay, *Tom20-KD* Tom20 knock-down, *siRNA* small interfering ribonucleic acid, *SD* standard deviation, *ns* not significant

To clarify the relationship between iron ions and B cell pyroptosis after SCI, we used FeSO_4_ and CCCP, which induces oxidative stress to simulate SCI, to interfere with B cell processes. B cells exhibited obvious characteristics of pyroptosis after iron–CCCP stimulation, such as cell swelling (Fig. 4a), decreased cell viability (Fig. 4b), and increased lactate dehydrogenase (LDH) release (Fig. 4c). ELISAs detected upregulated levels of inflammatory cytokines (MCP-1) in the supernatant of B cells stimulated by iron–CCCP (Fig. 4d). Western blot analysis revealed an upregulation of pyroptosis-related proteins of the Tom20–Bax–caspase–GSDME pathway in B cells stimulated by iron–CCCP (Fig. 4e). To verify the key role of Tom20, we used small interfering ribonucleic acid (siRNA) transfection to knock down Tom20 (Tom20-KD) in B cells (Fig. 4f). The results showed a reversal of the characteristics of pyroptosis in B cells stimulated by iron-CCCP, such as reduced cell swelling (Fig. 4g), increased cell viability (Fig. 4h), and decreased LDH release (Fig. 4i). ELISA detection revealed a downregulation of the levels of inflammatory cytokines (MCP-1) in the supernatant of Tom20-KD B cells (Fig. 4j). Western blot detection revealed no change in the expression of pyroptosis-related proteins in Tom20-KD B cells after iron-CCCP stimulation (Fig. 4k). Therefore, we believe that iron ions and Tom20 play key roles in driving B-cell pyroptosis. In addition, we co-cultured B cells with spinal cord neurons (Fig. 4l) indirectly and found that the supernatant of B cells stimulated by iron–CCCP increased neuronal apoptosis, while the supernatant of B cells of Tom20-KD reversed this phenomenon (Fig. 4m–p). This suggests that knocking down Tom20 may have a protective effect on neurons.

### Iron ions aggravate CCCP-induced human B lymphocytoma pyroptosis via activation of the Tom20–Bax–caspase–GSDME pathway

**Fig. 5:**
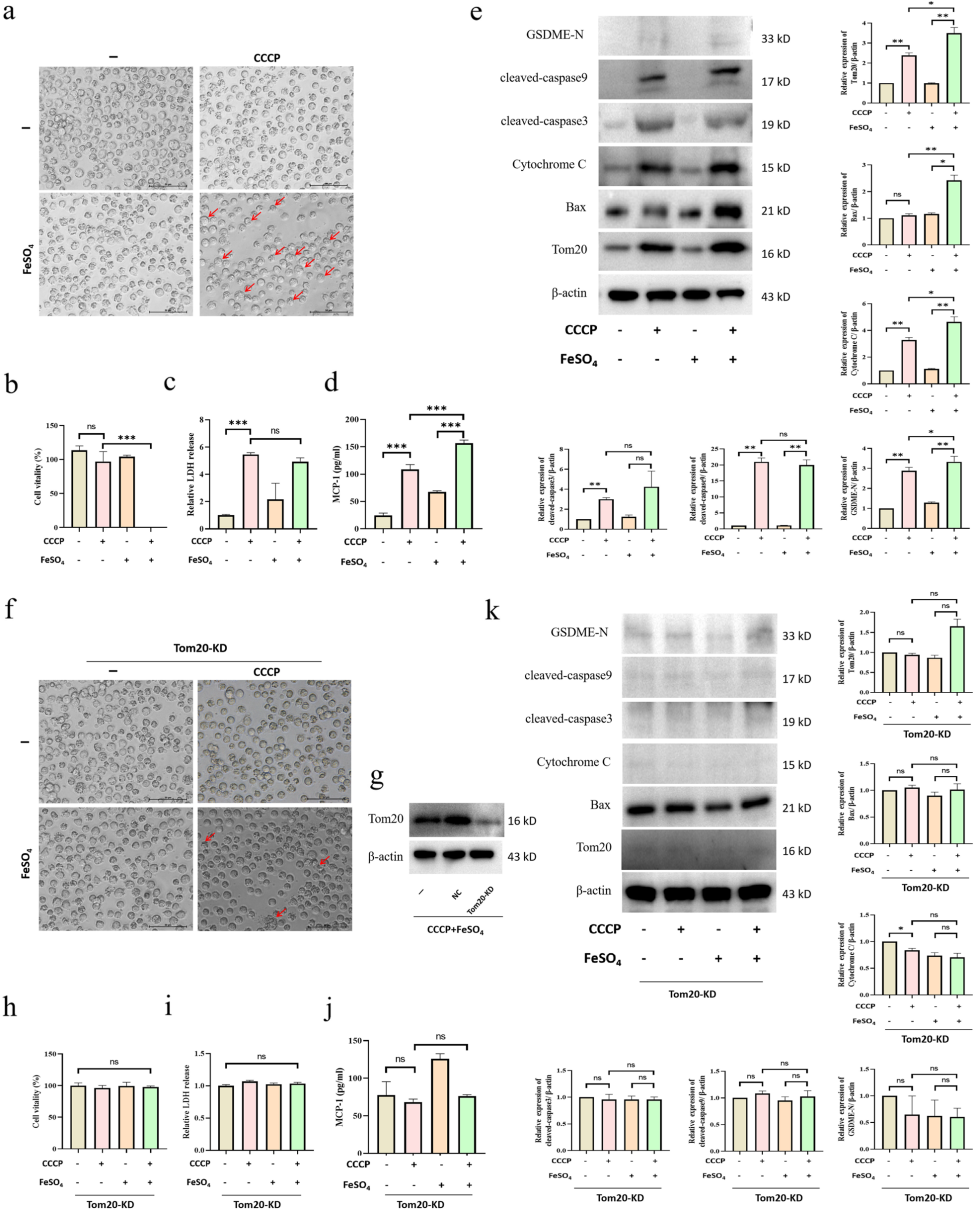
Effect of iron ions on CCCP-induced pyroptosis of Ramos cells. **a** FeSO_4_ and CCCP were used to induce interference in Ramos cells. After 24 h, the morphology of Ramos cells was observed. The red arrow indicates the presence of swollen B cells. **b** Detection of Ramos cell viability by CCK8. **c** Detection of LDH release from Ramos cell supernatants using an LDH kit. **d** Detection of the levels of MCP-1 in Ramos cell supernatants via ELISA. **e** Detection of the expression of Ramos cell pyroptosis-related proteins by western blot. **f**, **g** After Tom20-KD by siRNA transfection, the morphology of Ramos cells was observed. The red arrow indicates the presence of swollen Ramos cells. **h** Detection of Ramos cell viability by CCK8 after Tom20-KD. **i** Detection of LDH release from Ramos cell supernatants using an LDH kit after Tom20-KD. **j** Detection of the levels of MCP-1 in Ramos cell supernatants via ELISA after Tom20-KD. **k** Western blot detection of the expression of Ramos cell pyroptosis-related proteins after Tom20-KD. All data are expressed as the mean ± SD (*n* ≥ 3 replicates per group). ns *P* > 0.05, * *P* < 0.05, ** *P* < 0.01, *** *P* < 0.001. *CCCP* carbonyl cyanide m-chlorophenyl hydrazone, *CCK8* cell counting kit 8, *LDH* lactate dehydrogenase, *MCP-1* monocyte chemoattractant protein 1, *ELISA* enzyme-linked immunosorbent assay, *Tom20-KD* Tom20 knock-down, *siRNA* small interfering ribonucleic acid, *SD* standard deviation, *ns* not significant

To further verify the role of iron and Tom20 in human B lymphocytoma cell (Ramos) pyroptosis, we used the same methodology as in the mouse B cell experiments. FeSO_4_ and CCCP, which induce oxidative stress to simulate SCI, were used to interfere with Ramos cells. Ramos cells exhibited obvious characteristics of pyroptosis after iron-CCCP stimulation (Fig. 5a–c). It is worth noting that the viability of Ramos cells decreased significantly, although the release of LDH did not change significantly, possibly due to the fact that LDH had not been released from the cells. The effects of CCCP and FeSO_4_ on cytotoxicity were evaluated more comprehensively. Through ELISA detection, we also found upregulated levels of inflammatory cytokines (MCP-1) in the supernatant of Ramos cells stimulated by iron–CCCP (Fig. 5d). Western blotting analysis revealed an upregulation of pyroptosis-related proteins of the Tom20–Bax–caspase–GSDME pathway in Ramos cells stimulated by iron–CCCP (Fig. 5e). To verify the key role of Tom20, we used siRNA transfection to knock down Tom20 in Ramos cells (Fig. 5g). The results revealed a reversal of the characteristics of pyroptosis in Ramos cells stimulated by iron–CCCP (Fig. 5f, h, i). Through ELISA detection, a downregulation of the levels of inflammatory cytokines (MCP-1) was observed in the supernatant of Ramos cells after Tom20-KD (Fig. 5j). Western blotting analysis revealed no change in the expression of pyroptosis-related proteins in Tom20-KD Ramos cells stimulated with iron-CCCP (Fig. 5k). Therefore, we believe that iron ions and Tom20 play key roles in Ramos cell pyroptosis.

### Deferoxamine (DFO) can reduce inflammation and promote repair after SCI

**Fig. 6:**
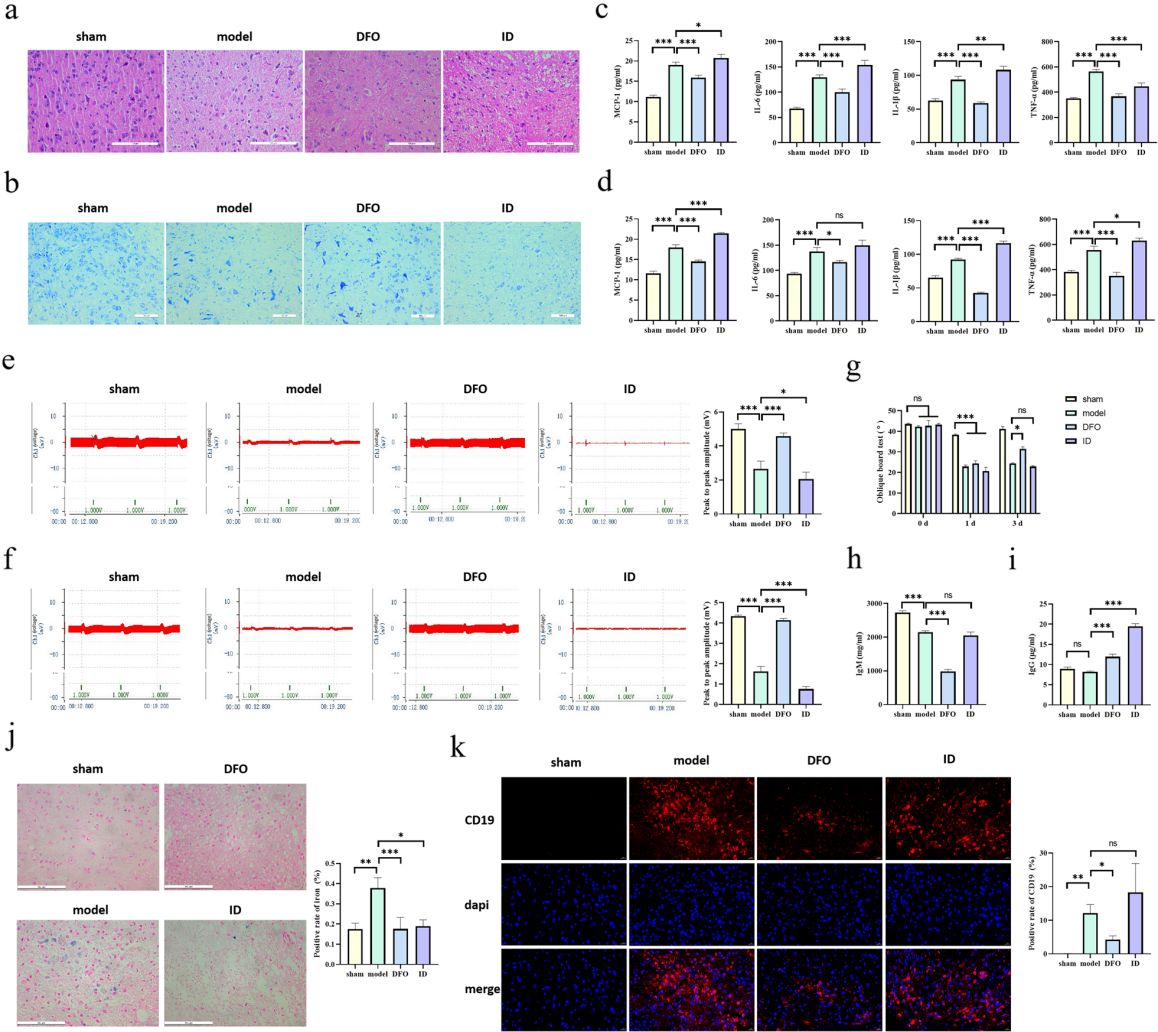
The effect of iron ions on repair processes after SCI. **a**, **b** After the establishment of the mouse SCI model, injured spinal cords were collected for HE staining to detect the overall structure and inflammatory infiltration and for Nissl staining to assess neuronal morphology and the intracellular condition. **c** Changes in expression of MCP-1, IL-6, IL-1β, and TNF-α in serum samples detected via ELISA. **d** Changes in expression of MCP-1, IL-6, IL-1β, and TNF-α in injured spinal cord tissue detected via ELISA. **e**–**g** The nerve evoked potential and electromyography, as well as oblique board test, were performed in different groups, and the changes in neuromotor function were observed. **h**, **i** The levels of IgM and IgG in serum were detected by ELISA. **j** The level of iron ion in the spinal cord tissue was detected by Prussian blue staining. **k** The distribution of B cells in the spinal cord tissue was detected by immunofluorescence. CD19^+^ was red, dapi was blue, and merge was combined. All data are expressed as the mean ± SD (*n* ≥ 3 replicates per group). * *P* < 0.05, ** *P* < 0.01, *** *P* < 0.001. *SCI* spinal cord injury, *HE* hematoxylin & eosin, *MCP-1* monocyte chemoattractant protein 1, *IL-6* interleukin 6, *IL-1β* interleukin 1 beta, *TNF-α* tumor necrosis factor alpha, *IgG* immunoglobulin G, *IgM* immunoglobulin M, *ELISA* enzyme-linked immunosorbent assay, *SD* standard deviation

To further explore the effect of iron ions on SCI, we used DFO (an iron chelator for the treatment of iron overload) and iron dextran (ID) (an iron supplement for the treatment of iron deficiency) to interfere with SCI processes in mice. Three days after SCI, pathological changes in cross-sectional areas of the injured spinal cord were observed by HE and Nissl staining. The staining revealed a disordered arrangement of nerve fibers, infiltration of a large number of inflammatory cells, and necrotic pyknosis of some neurons in the model group; these phenomena were alleviated in the DFO group and aggravated in the ID group (Fig. 6a, b), which was consistent with previous research results [21, 25]. Subsequently, we detected the levels of inflammatory cytokines (MCP-1, IL-6, IL-1β, and TNF-α) in serum and injured spinal cord tissue samples by ELISA. We found that the level of inflammatory cytokines increased significantly after SCI; however, DFO treatment decreased the level of inflammatory cytokines, whereas ID treatment aggravated the inflammatory reaction (Fig. 6c, d). The results of neuroelectrophysiology and oblique board test showed that DFO could significantly improve the motor function of mice with SCI (Fig. 6e–g). Prussian blue staining showed that the level of iron ion in spinal cord tissue of mice increased significantly after SCI, while DFO could significantly decrease the level of iron ion (Fig. 6j). Of note, ID also decreased the level of iron in the spinal cord tissue. We suspect that this may be related to the inflammatory stress response of the body. In addition, we detected the IgM and IgG levels in serum and observed the distribution of B cells in the spinal cord tissue by immunofluorescence. DFO significantly reduced the number of B cells in the spinal cord tissue and promoted the recovery of blood-spinal cord barrier (Fig. 6h, i, k). Therefore, DFO may reduce the inflammatory response by downregulating the level of iron ions and promoting repair mechanisms after SCI.

### DFO may reduce the inflammatory response after SCI by inhibiting Tom20-Bax-caspase-GSDME-induced B cell pyroptosis

**Fig. 7:**
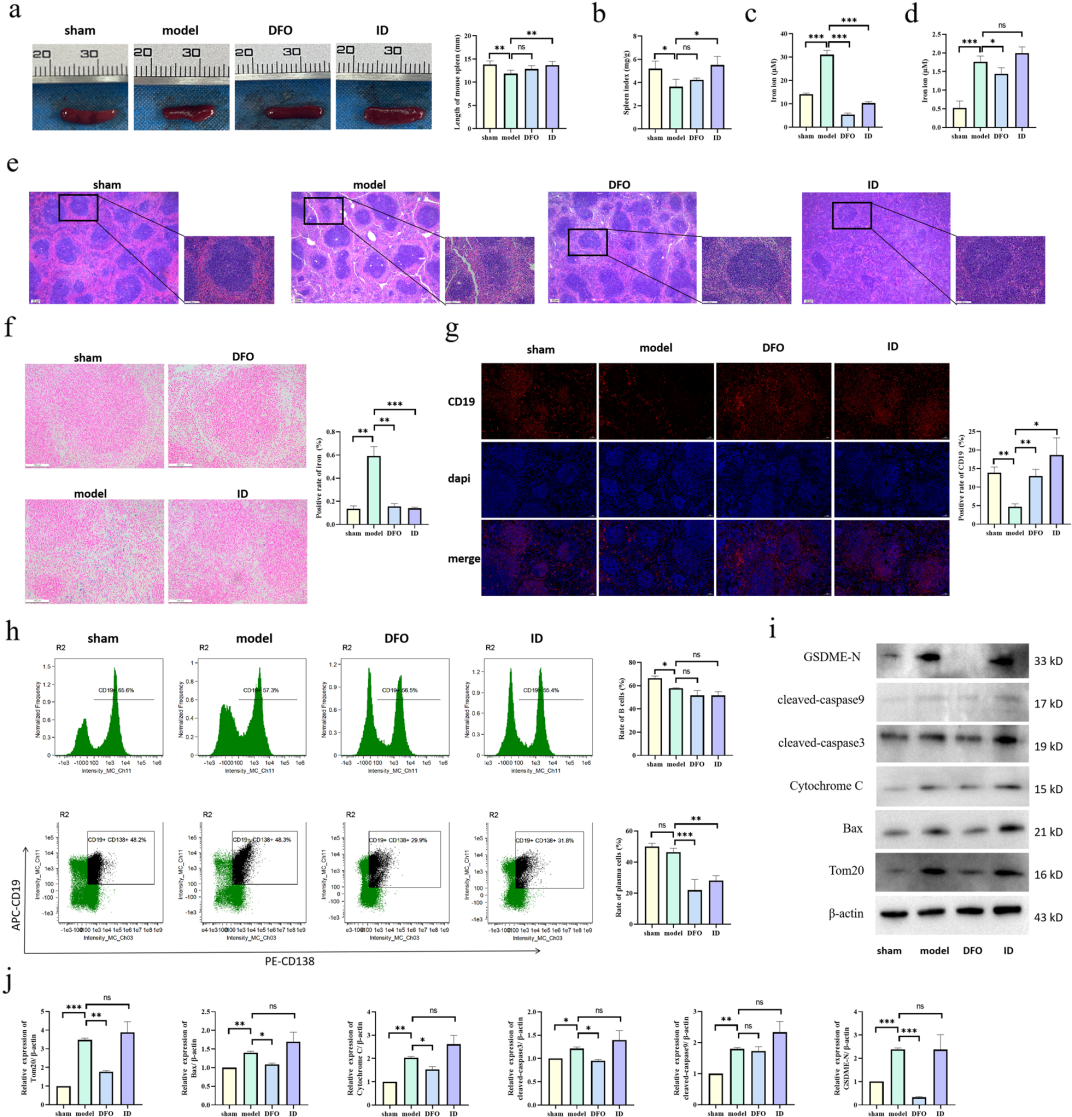
Specific mechanism through which iron ions affect repair processes after SCI. **a**, **b** The spleen of mice 3 days after SCI was observed, and the spleen length and organ index of different groups were compared. **c** Three days after SCI, serum samples were collected to detect the concentration of iron ions. **d** Three days after SCI, B cell lysates were collected to detect the concentration of iron ions. **e** The pathological changes of the spleen were detected by HE staining. **f** The level of iron ion in the spleen was detected by Prussian blue staining. **g** The distribution of B cells in the spleen was detected by immunofluorescence. CD19^+^ was red, dapi was blue, and merge was combined. **h** Lymphocytes were isolated from the spleen of mice three days after SCI and detected via flow cytometry (B cells were CD19^+^, and plasma cells were CD19^+^ and CD138^+^). **i**, **j** Detection of Tom20-Bax-caspase-GSDME pathway-related protein expression in B cells by western blot. All data are expressed as the mean ± SD (*n* ≥ 3 replicates per group). ns *P* > 0.05, * *P* < 0.05, ** *P* < 0.01, *** *P* < 0.001. *SCI* spinal cord injury, *HE* hematoxylin & eosin, *CD* cluster of differentiation, *GSDME* gasdermin E, *SD* standard deviation, *ns* not significant

To clarify the specific mechanism through which iron ions affect repair processes after SCI, we first observed the size and morphology of the spleen of mice. The length of the spleen of the model group became smaller, the color was darker, and the spleen index of the model group decreased significantly. Compared with the model group, no significant difference was observed in the DFO group, but the spleen length and spleen index in the ID group were significantly increased (Fig. 7a, b). Then we detected the levels of iron ions in serum samples and splenic B cells. The level of iron ions in the serum and splenic B cells increased significantly after SCI, while DFO treatment reduced the level of iron ions (Fig. 7c, d). In addition, Prussian blue staining showed that similar results in the level of iron ion in the spleen (Fig. 7f). Notably, ID could not increase the levels of iron ion in serum and spleen, consistent with the results of iron ion in the spinal cord tissue. The pathological changes of the spleen in mice were observed by HE staining and immunofluorescence. We found that the white pulp in the model group was atrophied, the structure was destroyed, and the number of B cells decreased. Compared with the model group, the spleen structure of the DFO group was significantly improved and the number of B cells was significantly increased (Fig. 7e, g). Of note, the spleen structure of the ID group was destroyed, and the number of B cells increased, showing pathological tissue proliferation. Flow cytometry experiments revealed that DFO decreased the level of B cell differentiation (Fig. 7 h) after SCI. The comprehensive analysis shows that DFO can affect the proliferation and differentiation of B cells after SCI, although the effects may be dependent on the timing of the intervention time and the concentration, resulting in possible failure to induce significant recovery of the spleen. Finally, we detected the expression of proteins related to the Tom20 pathway by western blotting and found that DFO could inhibit B cell pyroptosis induced by the Tom20-Bax-caspase-GSDME pathway after SCI (Fig. 7i, j). Therefore, DFO may downregulate the level of iron ions to inhibit the Tom20-Bax-caspase-GSDME pathway, reduce B cell pyroptosis, control the inflammatory response, and promote repair after SCI.

## Discussion

SCI is a process involving multiple forms of cellular degeneration caused by trauma. The inflammatory and immune responses influence each other, and the pathological mechanisms are complex and modifiable. The immune inflammatory response plays an important role in the occurrence and development of SCI; however, the beneficial and detrimental changes remain controversial [16]. Many studies have reported the role of neutrophils, macrophages, and T lymphocytes in the immune inflammatory response after SCI [26–30], although little is known about the role of B lymphocytes in these processes [31]. Some studies have shown that B cell deficiency can significantly reduce the degree of SCI [18], whereas Ig injection can exert a neuroprotective effect [19, 32]. These seemingly contradictory effects suggest that there may be an unknown pathological mechanism driving such changes.

First, we established a mouse model of SCI and found that the spleens of mice were atrophied and exhibited evidence of blood stasis. The ELISA and flow cytometry results demonstrated that SCI could downregulate the level of IgM in serum and decrease the number of B cells in the spleen. These results confirmed the presence of immunosuppression after SCI, consistent with the results of previous studies [16]. We used 4D label-free quantitative proteomics to analyze splenic B cells after SCI and found that SCI significantly upregulated the expression of Tom20 in B cells, a protein that is related to oxidative stress and the immune inflammatory response, with molecular action sites within the mitochondria and cell membrane. Subsequently, we verified the high expression of Tom20 using western blotting, which has not been reported in previous studies. It is worth mentioning that other differentially expressed proteins screened by the proteomic analysis may also play an important role, such as Slc9a1, HMGN2, and Emc3.

Some studies have suggested that iron ions can induce cell pyroptosis through the Tom20 pathway [22], and the level of iron ions is significantly upregulated after SCI [24]. Therefore, we also measured the level of iron ions after SCI and found that their levels were significantly increased in both the serum samples and B cells. The levels of expression of proteins related to the Tom20 pyroptosis pathway were detected via western blotting, which revealed that the Tom20–Bax–caspase–GSDME pathway was activated and induced B cell pyroptosis after SCI. To date, most studies that have investigated iron overload after SCI have focused on the mechanism of ferroptosis, such as that induced by iron overload in neurons after SCI [24, 33–35]. In addition, cell death is accompanied by the release of many inflammatory factors, which seriously affect the ability to repair damage after SCI [36]. In this study, we demonstrated a link between the accumulation of iron and B cell pyroptosis after SCI, which may represent a novel immune inflammatory response mechanism.

To clarify the relationship between iron ions and B cell pyroptosis after SCI, we used FeSO_4_ and CCCP, which induce oxidative stress to simulate SCI conditions [37], to interfere with B cells in mice and human B lymphocytoma cells, respectively. Both cell types exhibited obvious characteristics of pyroptosis after iron–CCCP stimulation, such as cell swelling, decreased cell viability, and increased LDH release. ELISA detection also revealed upregulated levels of inflammatory cytokines (MCP-1) in the supernatant of cells stimulated by iron–CCCP, which could explain why the absence of B cells can promote repair mechanisms after SCI. The western blot analysis revealed upregulation of the pyroptosis-related proteins of the Tom20–Bax–caspase–GSDME pathway in the two cell types after iron–CCCP stimulation, a phenomenon that was reversed by Tom20-KD with siRNA. Pyroptosis is a form of programmed cell death in which caspase-3, which is activated by certain stimuli, cleaves GSDME and releases its pore-forming domain (PFD) to generate pores, resulting in cell swelling and membrane rupture [38, 39]. Here, we verified that iron ions aggravate CCCP-induced B-cell pyroptosis by activating the Tom20–Bax–caspase–GSDME pathway. However, the gasdermin D (GSDMD) pathway is another mechanism of interest [40], and its relationship with iron-induced B pyroptosis warrants further exploration.

Many studies have shown that DFO can promote recovery after SCI by reducing the level of iron ions [21, 41–44], although the precise details remain unclear. We used DFO to interfere with the pathophysiology of SCI in mice and found that although B cells exhibited iron accumulation after SCI, DFO administration attenuated these changes in B cells and reduced the secretion of many inflammatory factors. Through western blot analysis, we found that DFO can inhibit B cell pyroptosis induced by the Tom20–Bax-caspase-GSDME pathway after SCI, which may be a new mechanism by which DFO promotes repair after SCI.

ID is a commonly used clinical iron supplement, which can significantly increase iron levels in serum [45]. The results of in vitro experiments showed that ID significantly aggravated the pathological changes of the spinal cord and spleen, increased the expression of many inflammatory factors, and decreased the neurological function of mice with SCI. Western blotting revealed that ID significantly promoted the occurrence of B cell scorch death in mice with SCI. However, the spleen index and length of mice, as well as number of B cells in the spleen, in the ID group were significantly increased. At the same time, we found that the concentration of iron in the serum of mice in the ID group decreased significantly, with no significant difference in the concentration of iron in B cells. The results of Prussian blue staining also revealed no iron accumulation in the spinal cord and spleen of the ID group, which may be related to the inflammatory stress response of the body. Following SCI in mice, iron overload occurs, leading to splenic dysfunction. Administration of ID may activate the pathway that inhibits iron levels, aggravate the inflammatory response, and lead to increased pathological edema of the spleen. In addition, ID may promote the metabolic excretion of iron ions or other forms of transfer in mice with SCI, which forms the direction of follow-up research.

In summary, we found that iron overload activates the Tom20–Bax–caspase–GSDME pathway after SCI, induces B cell pyroptosis, promotes inflammation, and aggravates the changes caused by SCI. This may represent a novel mechanism through which the immune inflammatory response is induced after SCI and may provide a new key target for the treatment of SCI.

## Supplementary Information

**Additional file 1.** Experimental procedures of 4D label-free quantitative proteomics.

## Data Availability

All relevant data are available and can be obtained upon request from the corresponding authors.
