# Supplementary material for: Iron induces B cell pyroptosis through Tom20–Bax–caspase–gasdermin E signaling to promote inflammation post-spinal cord injury
Source: J Neuroinflammation. 2023 Jul 22;20:171. doi: 10.1186/s12974-023-02848-0 (PMC10362643; doi:10.1186/s12974-023-02848-0)
Supplement: Supplementary file 1 — Additional file 1. Experimental procedures of 4D label-free quantitative proteomics. [file 12974_2023_2848_MOESM1_ESM.docx]

**Experimental procedures**

**1.1 Sample Preparation**

The method was determined by the project proposal or preliminary experiment report.

i. TCA/Acetone Precipitation and SDT Lysis[1]:

Application: Plant tissues (roots, stems, leaves, etc), hard tissues (skin, cartilage, hair, etc), fungi.

The samples were frozen in liquid nitrogen and ground with a pestle and mortar. 5 times volume of TCA/acetone (1:9) was added to the powder and mixed by vortex. The mixture was placed at -20°C for 4h, and centrifuged at 6000g for 40 min at 4°C. The supernatant was discarded. The pre-cooling acetone was added and washed for three times. The precipitation was air dried. 30 times volume of SDT buffer (4%SDS, 100mM Tris-HCl, pH 7.6) was added to 20-30 mg powder, mixed and boiled for 5 min. The lysate was sonicated and then boiled for 15 min. After centrifuged at 14000g for 15 min, the supernatant was filtered with 0.22 µm filters. The filtrate was quantified with the BCA Protein Assay Kit (P0012, Beyotime). The sample was stored at -80 °C.

ii. Homogenate and SDT Lysis[2]:

Application: Tender tissues (brain, liver, muscle, etc), mollusk, microorganism, etc.

SDT buffer was added to the sample, and transferred to 2 ml tubes with amount quartz sand. The lysate was homogenized by MP Fastprep-24 Automated Homogenizer (6.0M/S, 30s, twice). The homogenate was sonicated and then boiled for 10 min. After centrifuged at 14000g for 15 min, the supernatant was filtered with 0.22 µm filters. The filtrate was quantified with the BCA Protein Assay Kit (P0012, Beyotime). The sample was stored at -80 °C

iii. SDT Lysis[3]:

Application: Cell, protein powder, body fluid, concentrated fermentation broth, cell secretion, etc.

SDT buffer (4%SDS, 100mM Tris-HCl, pH 7.6) was added to the sample. The lysate was sonicated (this step can be skipped for protein solution) and then boiled for 10 min. After centrifuged at 14000g for 15 min, the supernatant was quantified with the BCA Protein Assay Kit (P0012, Beyotime). The sample was stored at -80 °C.

iv. Immunoaffinity Depletion of Serum High-Abundance Proteins:

Application: human, mouse or rat serum

Serum pools were depleted of most abundant proteins using Agilent Human 14 / Mouse 3 Multiple Affinity Removal System Column following the manufacturer’s protocol[4-6] (Agilent Technologies). The Human 14 column was applied for human, and Mouse 3 column was applied for mouse and rat. The 15 kDa ultrafiltration tube (Sartorius) was used for desalination and concentration of low-abundance components. One volume of SDT buffer (4%SDS, 100mM Tris-HCl, pH 7.6) was added, boiled for 10min and centrifuged at 14000g for 15 min. The supernatant was quantified with the BCA Protein Assay Kit (Bio-Rad, USA). The sample was stored at -80 °C.

**1.2 SDS-PAGE Separation**

20 µg of proteins for each sample were mixed with 6X loading buffer respectively and boiled for 5 min. The proteins were separated on 12% SDS-PAGE gel. Protein bands were visualized by Coomassie Blue R-250 staining.

**1.3 Filter-aided sample preparation (FASP Digestion)[4]**

50-200 μg of proteins for each sample were reduced with 100 mM DTT for 5 min at 100 °C. Then the detergent, DTT and other low-molecular-weight components were removed using UA buffer (8 M Urea, 150 mM Tris-HCl pH 8.5) by repeated ultrafiltration (Sartorius, 30 kD). Then 100 μl iodoacetamide (100 mM IAA in UA buffer) was added to block reduced cysteine residues and the samples were incubated for 30 min in darkness. The filters were washed with 100 μl UA buffer three times and then 100 μl 50 mM NH4HCO3 buffer twice.Finally, the protein suspensions were digested with 4 μg trypsin (Promega) in 40 μl 50 mM NH4HCO3 buffer overnight at 37 °C, and the resulting peptides were collected as a filtrate. The peptide segment was desalted by C18 column. The peptide content was estimated by UV light spectral density at 280 nm using an extinctions coefficient of 1.1 of 0.1% (g/l) solution that was calculated on the basis of the frequency of tryptophan and tyrosine in vertebrate proteins.

**2 Mass Spectrometry analysis**

Samples were analyzed on a nanoElute (Bruker, Bremen, Germany) coupled to a timsTOF Pro (Bruker, Bremen, Germany) equipped with a CaptiveSpray source. Peptides were separated on a 25cm X 75μm analytical column, 1.6μm C18 beads with a packed emitter tip (IonOpticks, Australia). The column temperature was maintained at 50°C using an integrated column oven (Sonation GmbH, Germany). The column was equilibrated using 4 column volumes before loading sample in 100% buffer A (99.9% MilliQ water, 0.1% FA) (Both steps performed at 800bar). Samples were separated at 300nl/min using a linear gradient as follows:

1.5 hours gradient: 2-22% buffer B for 75 min, 22-37% buffer B for 5 min, 37-80% buffer B for 5 min,hold in 80% buffer B for 5 min.

The timsTOF Pro (Bruker, Bremen, Germany) was operated in PASEF mode. Mass Range 100 to 1700m/z, 1/K0 Start 0.75 V⋅s/cm2 End 1.4 V⋅s/cm2, Ramp time 100ms, Lock Duty Cycle to 100%, Capillary Voltage 1500V, Dry Gas 3 l/min, Dry Temp 180°C, PASEF settings: 10 MS/MS scans (total cycle time 1.16sec), charge range 0-5, active exclusion for 0.5 min, Scheduling Target intensity 10000, Intensity threshold 2500, CID collision energy 20-59eV.

**3 Data Analysis**

The MS data were analyzed using MaxQuant software version 1.6.17.0. MS data were searched against the database( determined by project ). An initial search was set at a precursor mass window of 10 ppm. The search followed an enzymatic cleavage rule of Trypsin/P and allowed maximal two missed cleavage sites and a mass tolerance of 40ppm for fragment ions. Carbamidomethylation of cysteines was defined as fixed modification, while protein N-terminal acetylation and methionine oxidation were defined as variable modifications for database searching. The cutoff of global false discovery rate (FDR) for peptide and protein identification was set to 0.01. Protein aboundance was calculated on the basis of the normalized spectral protein intensity (LFQ intensity). Proteins which Fold change>2 or <0.5 and p value (Student’s t test) <0.05 were considered to be a differentially expressed proteins.

**4 Bioinformatics Analysis**

**4.1 Gene Ontology (GO) Annotation**

At first, all protein sequences were aligned to the database(determined by project) downloaded from NCBI (ncbi-blast-2.2.28+-win32.exe), only the sequences in top 10 and E-value<=1e-3 were kept. Secondly, the GO term (database version: go_201504.obo) of the sequence with top Bit-Score by Blast2GO was selected. Then, the annotation from GO terms to proteins was completed by Blast2GO Command Line. After the elementary annotation, InterProScan were used to search EBI database by motif and then add the functional information of motif to proteins to improve annotation. Then further improvement of annotation and connection between GO terms were carried out by ANNEX. Fisher's Exact Test were used to enrich GO terms by comparing the number of differentially expressed proteins and total proteins correlated to GO terms.

**4.2 KEGG Pathway Annotation**

Pathway analysis was performed using KEGG database. Fisher's Exact Test were used to identify the significantly enriched pathways by comparing the number of differentially expressed proteins and total proteins correlated to pathways.

**REFERENCE**

[1] MaxQuant enables high peptide identification rates, individualized p.p.b.-range mass accuracies and proteome-wide protein quantification. Cox, J. and M. Mann (2008).Nat Biotechnol 26(12): 1367-1372.

[2] Plant Proteomics: Methods and Protocols. Hervé Thiellement, Michel Zivy, Catherine Damerval, and Valerie Mechin, 2007. METHODS IN MOLECULAR BIOLOGY 355.

[3] Proteomic analysis of solid pseudopapillary tumor of the pancreas reveals dysfunction of the endoplasmic reticulum protein processing pathway. Zhu Y et al. Mol Cell Proteomics.2014. 13(10):2593-603.

[4] Universal sample preparation method for proteome analysis. Wisniewski, J. R., A. Zougman, et al. Nat Methods.2009. 6(5): 359-362.

[5] Agilent Human 14 Multiple Affinity Removal System Columns for the Fractionation of High-Abundant Proteins from Human Proteomic Samples. Agilent Technologies, Inc. 2007.

[6] Agilent Multiple Affinity Removal Columns – for Mouse Serum Proteins. Agilent Technologies, Inc. 2005.
